# *In vitro* and *in vivo* Study of Antifungal Effect of Pyrvinium Pamoate Alone and in Combination With Azoles Against *Exophiala dermatitidis*

**DOI:** 10.3389/fcimb.2020.576975

**Published:** 2020-10-23

**Authors:** Yi Sun, Lujuan Gao, Mingzhu Yuan, Lu Yuan, Ji Yang, Tongxiang Zeng

**Affiliations:** ^1^Department of Dermatology, Jingzhou Central Hospital, The Second Clinical Medical College, Yangtze University, Jingzhou, China; ^2^Department of Dermatology, Zhongshan Hospital Fudan University (Xiamen Branch), Xiamen, China; ^3^Department of Dermatology, Zhongshan Hospital Fudan University, Shanghai, China; ^4^Department of Clinical Medicine, Yangtze University, Jingzhou, China; ^5^Department of Pathology, Jingzhou Central Hospital, The Second Clinical Medical College, Yangtze University, Jingzhou, China

**Keywords:** *Exophiala dermatitidis*, pyrvinium pamoate, azole, antifungal, *Galleria mellonella*, synergy, fungi

## Abstract

Infections of *Exophiala dermatitidis* are often chronic and recalcitrant. Combination therapies with novel compounds and azoles could be an effective solution. Previously, we have demonstrated that pyrvinium pamoate exerted antifungal activity alone and favorable synergy with azoles against planktonic *E. dermatitidis*. Herein, the underlying antifungal mode of action were investigated. Pyrvinium alone showed sessile MIC50 (SMIC50) of 8->16 μg/ml against *E. dermatitidis* biofilms. However, synergism of PP with itraconazole, voriconazole, and posaconazole were observed against 16 (88.9%), 9 (50%), and 13 (72.2%) strains of *E. dermatitidis* biofilms. In accordance with *in vitro* susceptibilities, pyrvinium alone at concentration of 2 μg/ml resulted in significant growth restriction of planktonic *E. dermatitidis*. Pyrvinium alone resulted in reduction of biofilm formation. Higher concentration of pyrvinium was associate with more progressive reduction of biofilm mass. The *in vivo* activity of pyrvinium alone and combined with azoles was evaluated using *Galleria mellonella* model. Pyrvinium alone significantly improved the survival rate of larvae (*P* < 0.0001). The combination of pyrvinium and voriconazole or posaconazole acted synergistically *in vivo* (*P* < 0.05). Fungal burden determination revealed significant reduction of numbers of colony forming unit (CFU) in larvae treated with pyrvinium-itraconazole and pyrvinium-posaconazole compared to itraconazole or posaconazole alone group, respectively. The effect of pyrvinium on apoptosis, expression of *TOR* and *HSP90*, and drug efflux reversal were evaluated by PI/Annexin V staining, Real-Time Quantitative PCR and Rhodamine 6G assay, respectively. Pyrvinium alone or combined with azoles significantly (*P* < 0.05) increased late apoptosis or necrosis of *E. dermatitidis* cells. Pyrvinium combined with posaconazole significantly decreased the expression of *TOR* and *Hsp90* compared to posaconazole alone group (*P* < 0.05). Pyrvinium resulted in significant (*P* < 0.05) decrease of the efflux of Rhodamine 6G. These findings suggested pyrvinium could be a promising synergist with azoles. The underlying mechanisms could be explained by inducing apoptosis/necrosis, inhibition of drug efflux pumps, and signaling pathways related with stress response and growth control.

## Introduction

The genus of *Exophiala* are the most frequently associated black fungi in clinic (Kirchhoff et al., 2019). Among the genus, *Exophiala dermatitidis*, previously known as *Wangiella dermatitidis*, is most commonly found as a pathogen in the human host (Zeng et al., 2007; Kirchhoff et al., 2019). It causes a variety of disease in humans, including phaeohyphomycosis in immunosuppressed patients and fatal neurotropic infections in immunocompetent individuals (Li et al., 2011; Kirchhoff et al., 2019; Vila et al., 2019; Wang et al., 2019). The mortality rates of systemic and invasive infections are among the range of 25–80% (Kirchhoff et al., 2019). Additionally, *E. dermatitidis* has been frequently isolated as a pulmonary colonizer in cystic fibrosis patients and plays a vital role in clinical deterioration (Kondori et al., 2011; Martin Ramirez et al., 2019).

Prompt antifungal treatment is crucial to prevent life-threatening disease. Surgical resection serves as adjunctive therapy for local lesions. Apart from surgery, antifungal therapy alone or in combination is required. Monotherapy with azoles is the first-line treatment (Chowdhary et al., 2014). Amphotericin B is the second option for monotherapy. Although *in vitro* susceptibilities revealed favorable results of common antifungals (Badali et al., 2011; Sun et al., 2011), outcome rates with amphotericin B, voriconazole and itraconazole treatment were only 44, 50, and 71.4%, respectively (Revankar and Sutton, 2010; Kondori et al., 2011; Patel et al., 2013). Even with the combination modality of azoles and amphotericin B, the success rates are virtually similar to that of monotherapy (Patel et al., 2013). Therefore, novel agent for combination approaches is in desperate need.

Pyrvinium pamoate (PP), approved by FDA in 1955, is an antihelmitic agent used to treat strongyloidiasis and pinworm (*Enterobius vermicularis*) infections in humans (Beck et al., 1959; Wagner, 1963). Interestingly, PP has attracted considerable attentions for its potential antitumor properties in recent years (Momtazi-Borojeni et al., 2017). In addition, it has also been demonstrated that PP strongly inhibited the growth of aneuploid strain of *Candida albicans* (the isochromosome 5L strain), which was known to confer resistance to fluconazole, and enhanced the efficacy of fluconazole (Chen et al., 2015). In our previous study, we evaluated the *in vitro* efficacy of PP alone and in combination with azoles against planktonic *E. dermatitidis* by the checkerboard microdilution methods (Gao et al., 2018). Inspiringly, PP exerted antifungal activity alone (MIC 2 μg/ml) and favorable synergistic effects with azoles against *E. dermatitidis* (Gao et al., 2018).

In the present study, we further investigated the antimicrobial effects of PP on the growth of planktonic cells and biofilm of *E. dermatitidis*. In addition, the *in vivo* antifungal effects of PP alone and combined with azoles were investigated. Related antifungal mode of action were also explored, including the effect of PP alone and combined with azoles on apoptosis of *E. dermatitidis* cells, drug efflux pump activity, and gene expression of target of rapamycin (TOR) signaling pathway and calcineurin-Hsp90 pathway related genes.

## Materials and Methods

### Fungal Strains, Antifungals and Chemical Agents

The *E. dermatitidis* strains were grown on Sabouraud Dextrose Agar (SDA) at 37°C. All strains were clinical isolates. Fungal identification was determined by microscopic morphology and by molecular sequencing of the internal transcribed spacer (ITS) ribosomal DNA (rDNA), as required. For determination of *in vitro* susceptibilities, a total of 18 strains of *E. dermatitidis* were studied. *Candida parapsilosis* ATCC 22019 was included to ensure quality control.

Antifungals and chemical agents including PP, itraconazole, voriconazole, and posaconazole were purchased in powder form from Selleck Chemicals, Houston, TX, USA and diluted in dimethyl sulfoxide as stock solutions (6,400 μg/ml).

### *In vitro* Interactions of PP and Azoles Against *E. dermatitidis* Biofilm

The interactions between PP and azoles against biofilms of *E. dermatitidis* were tested via the microdilution chequerboard technique, adapted from the Clinical and Laboratory Standards Institute broth microdilution method M38-A2 (Clinical Laboratory Standards Institute, 2008) and assessed by the XTT {2,3-bis-(2-methoxy-4-nitro-5-sulfophenyl)-2H-tetrazolium-5-carboxanilide} based colorimetric assay (Ramage et al., 2001). The working concentration ranges of tested agents were 0.5–16 μg/ml for PP and 0.5–64 μg/ml for azoles, respectively. The biofilms were prepared as described (Gao et al., 2016). *E. dermatitidis* strain BMU00034 was used as a reference isolate. *E. dermatitidis* conidia were collected from Sabouraud Dextrose broth after incubation at 28°C in a shaker at 180 rpm for 48 h and washed with phosphate-buffered saline (PBS). The conidia were resuspended in RPMI-1640 and which was then adjusted to the final concentration of 1 × 10^7^ spores/ml. Subsequently, the suspension was added into the 96-well plate with 200 μl in each cell and incubated at 37°C for 72 h. The media were then carefully extracted without disturbing the biofilm. The 96-well plate was washed with sterile PBS for three times to remove detached spores (Pierce et al., 2008). The 96-well plate containing prepared biofilm was then inoculated with 50 μl serially double-diluted PP in the horizontal direction and another 50 μl serially double-diluted azoles in the vertical direction. After incubation at 37°C for 48 h, 100 μl XTT/menadione solution was added in each well and then incubated for another 4 h. The working concentrations of XTT/menadione were 100 μg/ml XTT with 1 μM menadione. Subsequently, 80 μl of the colored supernatant from each well was removed and transferred into a new plate, and read at 490 nm. The SMIC50 was defined as the concentration at which a 50% decrease in optical density would be detected in comparison to the controls (Pierce et al., 2008). Drug combination interaction was classified on the basis of the fractional inhibitory concentration index (FICI). The FICI was calculated by the formula: FICI = (Ac/Aa) + (Bc/Ba), where Ac and Bc are the SMICs of antifungal drugs in combination, and Aa and Ba are the SMICs of antifungal drugs A and B alone (Tobudic et al., 2010). The FICI was classified as follows: FICI of ≤0.5, synergy; FICI of >0.5 to ≤4, no interaction (indifference); FICI of >4, antagonism (Odds, 2003). All tests were performed in triplicate.

### Effect of PP on the Growth of Planktonic *E. dermatitidis* Cells

The effect of PP on the growth of planktonic *E. dermatitidis* cells was evaluated by adding an 200 μl inoculum of conidia to RPMI-1640 containing PP at concentrations of 0, 2, and 4 μg/ml. The final concentration of conidia was 1 × 10^4^ spores/ml. Cells of *E. dermatitidis* (strain BMU00034) were incubated at 37°C for 36 h in a shaker at 100 rpm. Aliquots of each culture were collected after 0.5, 1, 2, 4, 6, 8, 10, 12, 24, and 36 h of incubation and inoculated evenly on Potato Dextrose Agar (PDA). The number of colony-forming unit (CFU) was determined after 72 h incubation at 37°C. All tests were performed in triplicate. Reductions in the growth curve of treated cells were determined relative to untreated cells.

### Effect of PP on *E. dermatitidis* Biofilm Formation

Methods used for *E. dermatitidis* biofilm formation in 24-well plates were performed as described above. In brief, *E. dermatitidis* conidia were collected from Sabouraud Dextrose broth after incubation at 28°C in a shaker at 180 rpm for 48 h and washed with phosphate-buffered saline (PBS). The conidia were resuspended in RPMI-1640 and adjusted to the final concentration of 1 × 10^7^ spores/ml. Then, a 1-ml aliquot of the suspension was added to designated wells of the microtiter plate with circular growth cover glasses (ϕ13 mm). Sterile RPMI-1640 was used as a control for a blank correction. To evaluate the effect of PP on the formation of *E. dermatitidis* biofilms, PP was added concomitantly to the conidia suspension in RPMI-1640 used for the biofilm formation to achieve final concentrations of 0, 2, 4, 8, 16, and 32 μg/ml. Cells were then incubated at 37°C for 72 h to allow the formation of biofilms. The supernatant was then discarded and the growth cover glasses were stained with calcofluor white. The morphology of *E. dermatitidis* biofilm were observed under fluorescence microscope. Experiments were performed in triplicate.

### *In vivo* Efficacy and Fungal Burden Determination

Efficacy of PP alone and combined with azoles in *G. mellonella* infected with *E. dermatitidis* was evaluated by survival assay as described previously (Maurer et al., 2015; Bakti et al., 2018), using sixth instar larvae (~300 mg, Sichuan, China). Groups of 20 larvae was maintained in wood shavings in the dark at room temperature before use. Suspensions of *E. dermatitidis* (strain BMU00034) that had been grown on SDA for 72 h at 37°C were harvested by gentle scraping of colony surfaces with sterile plastic loops, washed twice, and adjusted to 1 × 10^7^ spores/ml in sterile saline. A Hamilton syringe (25 gauge, 50 μL) was used to inoculate larvae with *E. dermatitidis* suspension and for introduction of treatments or control solutions via the last right proleg of the larvae. To determine the *in vivo* effects of PP alone and in combination with azoles against *E. dermatitidis*, a total of seven intervention therapy groups were included, namely PP treated group, ITC treated group, POS treated group, VRC treated group, PP with ITC treated group, PP with POS treated group, and PP with VRC treated group. Larvae were infected with 5 μL conidia suspension per larvae and injected with 5 μl tested agents (0.5 μg per agent) 2 h post-infection. The following control groups were included: larvae injected with sterile saline, larvae injected with conidia suspension, and untouched larvae. For survival studies, the death of larvae was monitored by visual inspection of their color (brown-dark/brown) each 24 h for 5 days. For tissue burden studies, 3 larvae from each group were selected with no discrimination at every 24 h, suspended in 1 ml of saline-ampicillin, and gently homogenized for a few seconds. The mix was 100 fold diluted with PBS buffer and 100 μl of the dilutions were inoculated on the SDA. The colonies were counted after 2 days of incubation at 37°C for 48 h. Experiment was repeated three times using larvae from different batches.

### Histological Study in *Galleria mellonella*

To evaluate the presence of *E. dermatitidis* in tissue of *G. mellonella*, larvae from different groups (see above) were collected after death. The larvae were preserved in 70% ethanol and further cut into 8-μm-thick sections via a cryostat. The samples were dried naturally at room temperature for 2 days, stained with Periodic Acid Schiff reagent (PAS), and dehydrated with increasing concentrations of ethanol and xylol (70, 80, 90, 96, and 100%). Finally, the samples were fixed in neutral balata and dried naturally at room temperature for 2 days. The stained tissues were observed under microscope. The experiment was repeated three times using larvae from different batches.

### Effect of PP Alone and Combined With Azoles on Apoptosis of *E. dermatitidis* Cells

Cell apoptosis was evaluated via Annexin V-EGFP apoptosis detection kit (Enogene, Nanjing, China). A total of 8 groups were set, namely control group, PP group, ITC group, VRC group, POS group, PP combined with ITC group, PP combined with VRC group, and PP combined with POS group. The working concentration of PP and azoles were 0.5 and 1 μg/ml, respectively. Cultures without drugs were served as controls. *E. dermatitidis* cells (strain BMU00034) were grown on PDA at 28°C for 3–5 days. Conidia suspension at a concentration of 1 × 10^6^ spores/ml were prepared and divide into 50 ml portions, which were subsequently incubated with tested agents for 6 h at 37°C under constant agitation (150 rpm). Conidia were then collected, washed, and resuspended with PBS. About 1–5 × 10^5^ cells were collected by centrifugation and resuspended in 500 μl of binding buffer. Next, 5 μl of Annexin V-EGFP and 5 μl of propidium iodide were added and incubated at room temperature for 10 min in the dark. Cells were quantified using a EpicsXL flow cytometer (Beckman Coulter, USA). Cell morphology was observed under fluorescence microscope. Experiments were performed in triplicate and for each sample, 10,000-events were collected and analyzed using CytExpert software (Beckman Coulter, CA, USA). Early apoptotic cells appeared in the annexin V+/PI– fraction, whereas late phase apoptotic cells or necrotic cells appeared in the annexin V+/PI+ fraction. Undamaged cells remained negative for both parameters, whereas cells damaged by scraping appeared in the annexin V–/PI + fraction.

### Effect of PP Alone and Combined With Azoles on the Expression of *TOR* and *HSP90*

mRNA expression of TOR signaling pathway and calcineurin-Hsp90 pathway related genes (*TOR* and *HSP90*) was measured by Real-Time Quantitative PCR (RT-qPCR). A total of 8 groups were set, namely control group, PP group, ITC group, VRC group, POS group, PP combined with ITC group, PP combined with VRC group, and PP combined with POS group. Cultures without drugs were served as controls. The working concentration of PP and azoles were 0.5 and 1 μg/ml, respectively. *E. dermatitidis* cells (strain BMU00034) were grown on PDA agar at 28°C for 3–5 days. Conidia suspension at a concentration of 1 × 10^6^ spores/ml were prepared and divide into 50 ml portions, which were subsequently incubated with tested agents for 6 h at 37°C under constant agitation (150 rpm). Total RNA was extracted from cells using E.Z.N.A. Fungal RNA Kit (Omega Bio-tek, GA, USA) and was transcribed to cDNA using Hifair® II 1st Strand cDNA Synthesis SuperMix for qPCR (gDNA digester plus) (YEASEN, Shanghai, China) in accordance with the manufacturer's instructions. The RT-qPCR was performed by using Hieff® qPCR SYBR-Green Master Mix (YEASEN, Shanghai, China) in ABI 7500 RT-PCR system. The primers used for RT-qPCR were showed in Table 1. The levels of each gene was calculated using the 2^−ΔΔCT^ method (Livak and Schmittgen, 2001). The relative expression level of *TOR* and *HSP90* was normalized to that of internal control *tubulin*. Experiments were performed in triplicate.

**Table 1:** The primers used for RT-qPCR.

**Primers**	**Sequences**
TORF	5′-CGAGCGGTGCAGGTGCTTGC-3′
TORR	5′-CCACACTCGTTGCCTGCGCG-3′
HSP90F	5′-TCATGATACGCTC-CATGTTG-3′
HSP90R	5′-GAGGAGCTCAACAAGACCAA-3′
tublinF	5′-GCACGTGAAATTGTTGAAAGG-3′
tublinR	5′-CAGGCTGGCCGCATTG-3′

### Effect of PP on the Efflux of Rhodamine 6G

The efflux of R6G was determined using previously published protocols (Natesan et al., 2013). Briefly, approximately 1 × 10^8^ spores/ml of *E. dermatitidis* (strain BMU00034) suspension were incubated in Sabouraud Dextrose broth at 37°C for 12 h. Conidia were centrifuged for 5 min, washed with PBS at 4°C and suspended in glucose-free PBS at a concentration of 1 × 10^8^ spores/ml. R6G (10 μM) with or without PP (2 μg/ml) were added to the conidial suspensions and incubated for 60 min at 37°C under constant agitation (50 rpm) to allow R6G accumulation. The cells equilibrated with R6G were collected by centrifugation, washed twice with PBS and resuspended in glucose-free PBS. At 0, 5, 10, 15, 20, 25, 30, 35, 40, and 45 min time points, samples of 1 ml were withdrawn and centrifuged for 2 min. The supernatants were collected and transferred into 96-well microtiter plates. The absorbance of supernatants was measured at 533 nm. In order to assess the efflux of Rh6G, 4 groups were set in this experiment: growth control group (PP-free, glucose free), PP (2 μg/ml) without glucose group, PP (2 μg/ml) with glucose group, and glucose group (2% glucose was added to the supernatants at 10 min). The experiment was repeated on 3 independent occasions.

### Statistical Analysis

Data were presented as mean ± SEM. All experiments were performed in triplicate. Graphs production, data distribution and statistical analyses were performed using Graph Pad Prism 7. After ensuring data conformed to a normal distribution, before and after data transformation, analysis of variance (ANOVA) and *t*-tests were used to investigate significant differences between independent groups. The *G. mellonella* survival curves were analyzed by the Kaplan–Meier method. Differences were considered significant at *P* < 0.05.

## Results

### *In vitro* Interactions of PP and Azoles Against *E. dermatitidis* Biofilm

The SMIC50 ranges of individual tested agents were all 8->16 μg/ml for PP, 16–64 μg/ml for ITC, 8–16 μg/ml for VRC and 8–32 μg/ml for POS (Table 2). When PP was combined with azoles, the SMIC50 ranges decreased to 1–8 μg/ml for PP, 2–16 μg/ml for ITC, and 2–8 μg/ml for VRC and POS. Based on the FICIs calculated from SMIC50, favorable synergistic effects of PP-ITC, PP-VRC, and PP-POS combinations were observed against 16 (88.9%), 9(50%), and 13(72.2%) strains of *E. dermatitidis*, respectively.

**Table 2:** SMICs and FICIs results with combinations of PP with azoles against *E. dermatitidis* biofilms.

**Strain**	**SMIC50 (μg/ml)**				**SMIC50 [A/B(μg/ml)] (FICI**^**a**^**)**		
	**PP**	**ITC**	**VRC**	**POS**	**PP/ITC**	**PP/VRC**	**PP/POS**
BMU00028	16	32	16	16	4/4 (S)	2/4 (S)	4/8 (I)
BMU00029	>16	32	8	8	8/8 (S)	4/8 (I)	2/4 (I)
BMU00030	8	32	16	16	2/16 (I)	2/4 (S)	1/4 (S)
BMU00031	16	64	8	16	4/16 (S)	4/8 (I)	2/4 (S)
BMU00034	16	32	16	8	4/8 (S)	4/4 (S)	4/2 (S)
BMU00035	8	64	16	16	4/16 (I)	4/8 (I)	4/2 (I)
BMU00036	8	32	8	8	2/8 (S)	4/4 (I)	2/2 (S)
BMU00037	16	32	16	16	4/8 (S)	4/2 (S)	4/4 (S)
BMU00038	16	16	16	16	4/2 (S)	4/4 (S)	4/4 (S)
BMU00039	>16	32	8	8	8/4 (S)	4/2 (S)	4/8 (I)
BMU00040	8	32	8	16	2/8 (S)	2/8 (I)	4/4 (I)
BMU00041	16	32	8	8	4/8 (S)	2/8 (I)	4/2 (S)
109140	>16	32	16	16	4/8 (S)	4/4 (S)	4/2 (S)
109144	16	32	16	16	4/4 (S)	4/4 (S)	4/2 (S)
109145	>16	16	16	16	4/2 (S)	4/8 (I)	4/4 (S)
109148	16	32	16	16	4/16 (I)	8/8 (I)	2/4 (S)
109149	16	32	16	16	4/4 (S)	4/4 (S)	2/2 (S)
109152	16	64	16	32	8/16 (I)	4/8 (I)	4/4 (S)

a *S, synergy (FICI of ≤ 0.5); I, no interaction (indifference) (0.5 < FICI ≤ 4)*.

### Effect of PP on the Growth of Planktonic *E. dermatitidis* Cells

**Figure 1:**
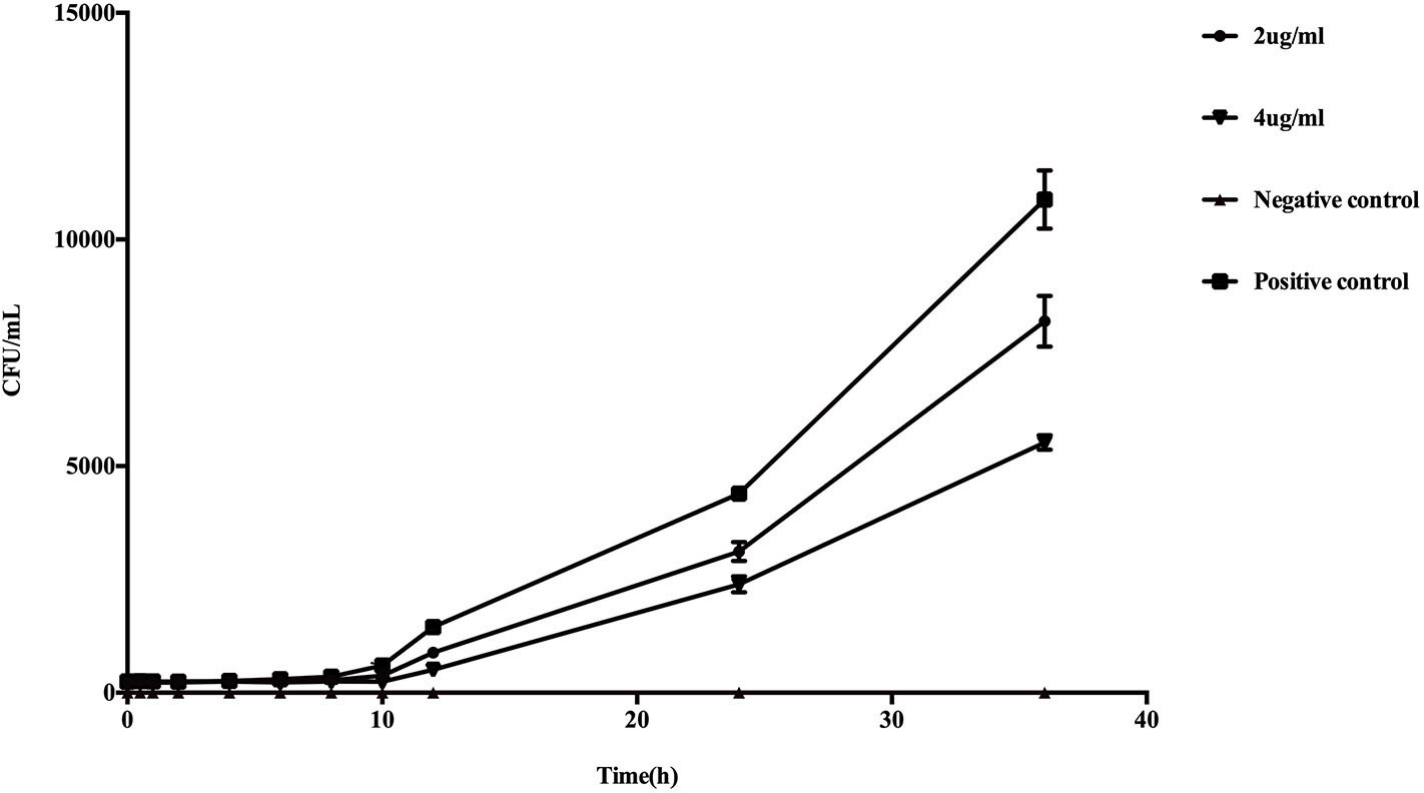
*In vitro* effect of PP on the growth of planktonic *E. dermatitidis*. Growth curves began to diverge after 6 h of incubation. After 10 h, changes in the growth rates were statistically different (*P* < 0.05) in PP group (both 2 and 4 μg/ml) compared with conidia culture without PP. The greatest reduction in growth rate was seen with 4 μg/ml PP (49.3%) compared with untreated control.

Higher concentrations of PP resulted in more significant inhibition of the growth of planktonic *E. dermatitidis* (Figure 1). Following 36 h of incubation in liquid culture, restriction of growth was statistically significant at PP concentration of 2 μg/ml (*P* < 0.05) and was maximally reduced by 49.3% at 4 μg/ml.

### Effect of PP on *E. dermatitidis* Biofilm Formation

**Figure 2:**
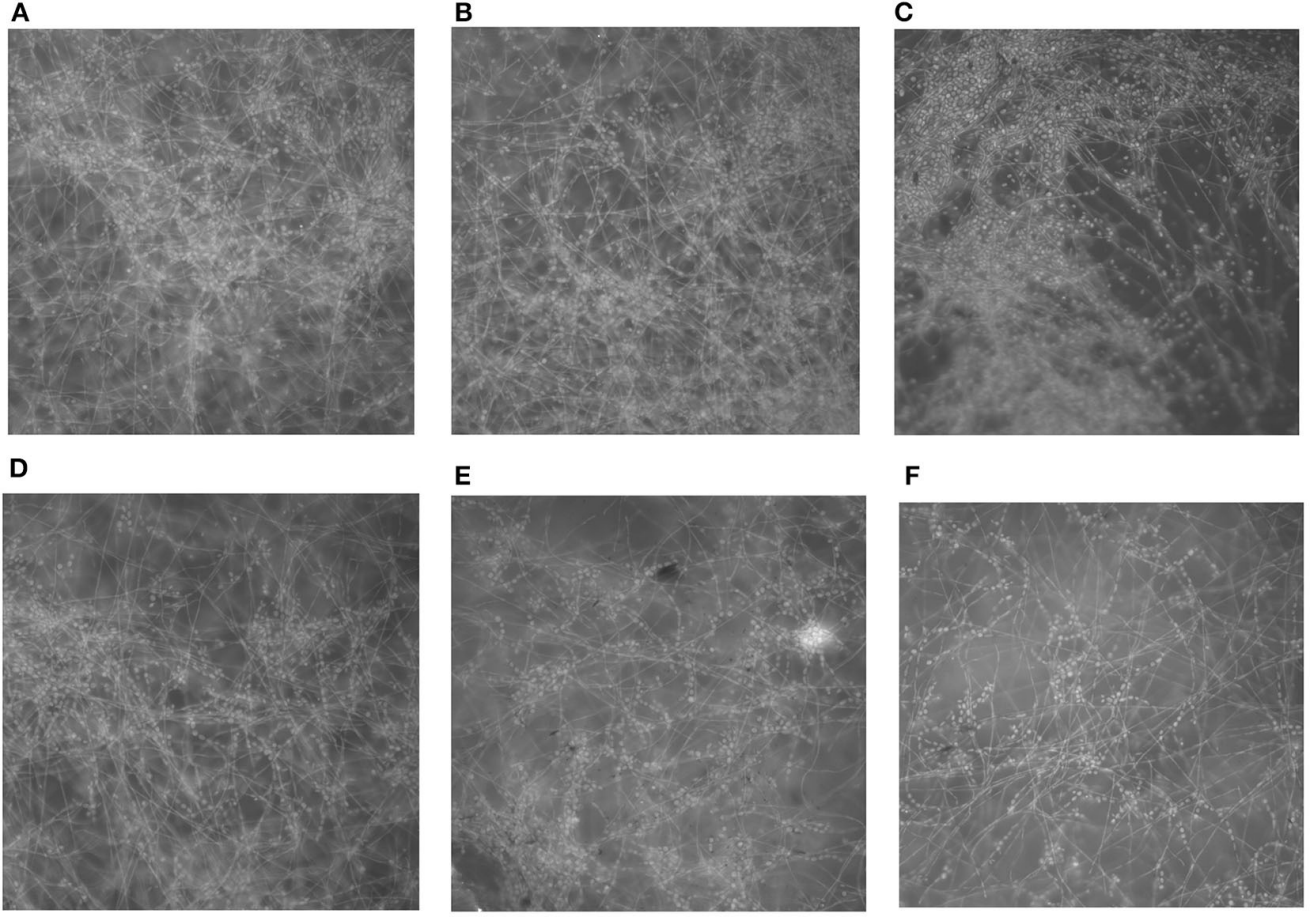
*In vitro* effect of PP on the formation of *E. dermatitidis* biofilms. **(A)** biofilm without PP; **(B–F)** biofilm with PP at doubling concentrations of from 2 to 32 μg/ml. Biofilms formed in the presence of PP showed a progressive decrease in biofilm mass with the increasing of PP concentration.

Light microscopy of the biofilms formed in the presence of PP revealed a clear reduction in biofilm mass with increasing concentrations of PP (Figure 2). At 16 and 32 μg/ml of PP, there was a marked decrease in the density of the biofilms.

### *In vivo* Effect of PP Alone and Combined With Azoles in *Galleria mellonella*

**Figure 3:**
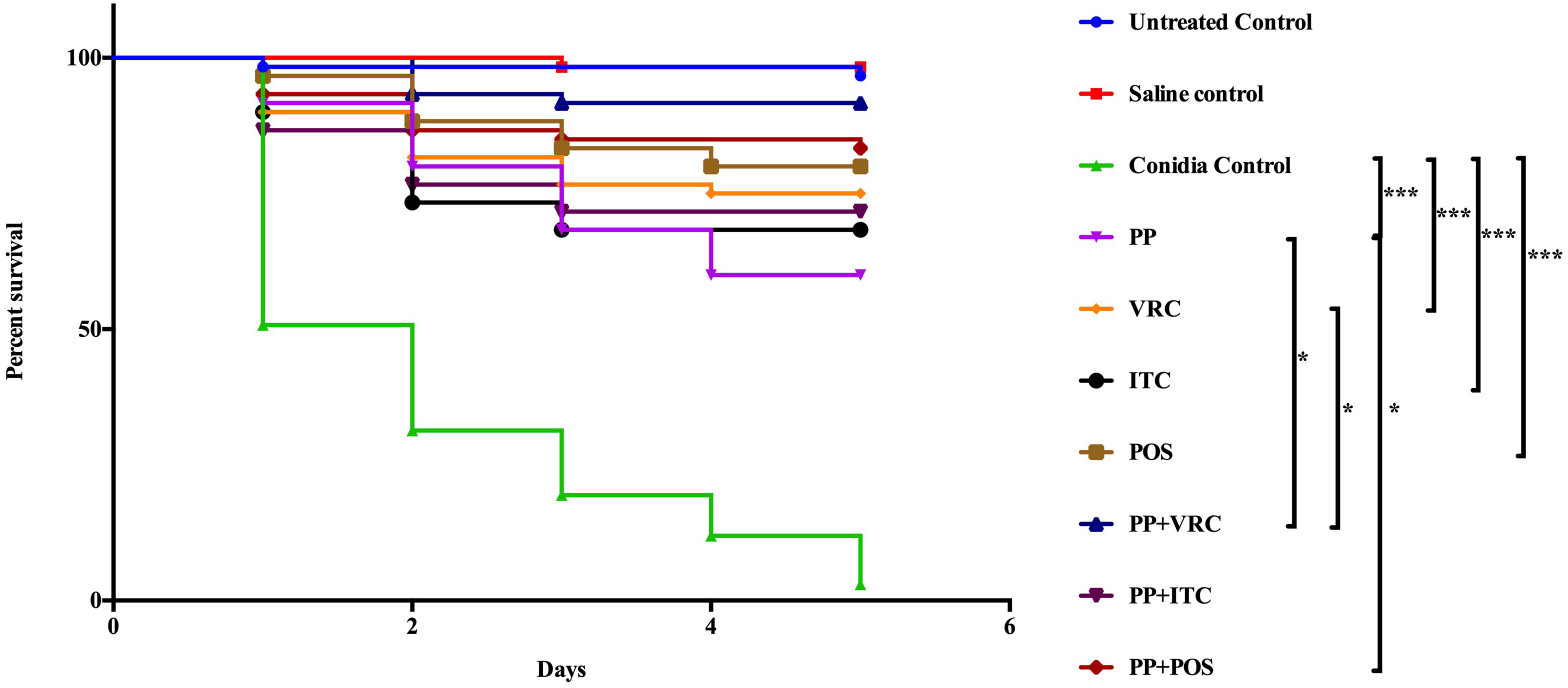
Survival curve of different treatment on *G. mellonella* infected with *E. dermatitidis*. The curves were consisted of sterile saline group, conidia only group, untouched growth control group, PP treated group, ITC treated group, POS treated group, VRC treated group, PP with ITC treated group, PP with POS treated group, and PP with VRC treated group. Treatment with PP or azoles alone significantly (*P* < 0.0001) prolonged the survival of larvae compared to control groups that received conidial suspension only. In groups that received PP combined with VRC, survival of larvae was significantly (*P* < 0.05) prolonged compared to groups that received PP only or VRC only. Treatment with PP and POS also had a significant (*P* < 0.05) positive effect on survival compared to groups received PP alone. (***P < 0.001; *P < 0.05).

Treatment with PP or azoles alone significantly (*P* < 0.0001) prolonged the survival of larvae compared to control groups that received conidial suspension only (Figure 3). In groups that received PP combined with VRC, survival of larvae was significantly (*P* < 0.05) prolonged compared to groups that received PP or VRC only (Figure 3). Treatment with combination of PP and POS also had a significant (*P* < 0.05) positive effect on survival compared to groups received PP alone (Figure 3). PP combined with ITC seemed to have a positive effect on survival compared to PP or ITC alone, although statistically not significant (Figure 3).

### Fungal Burden Determination

**Figure 4:**
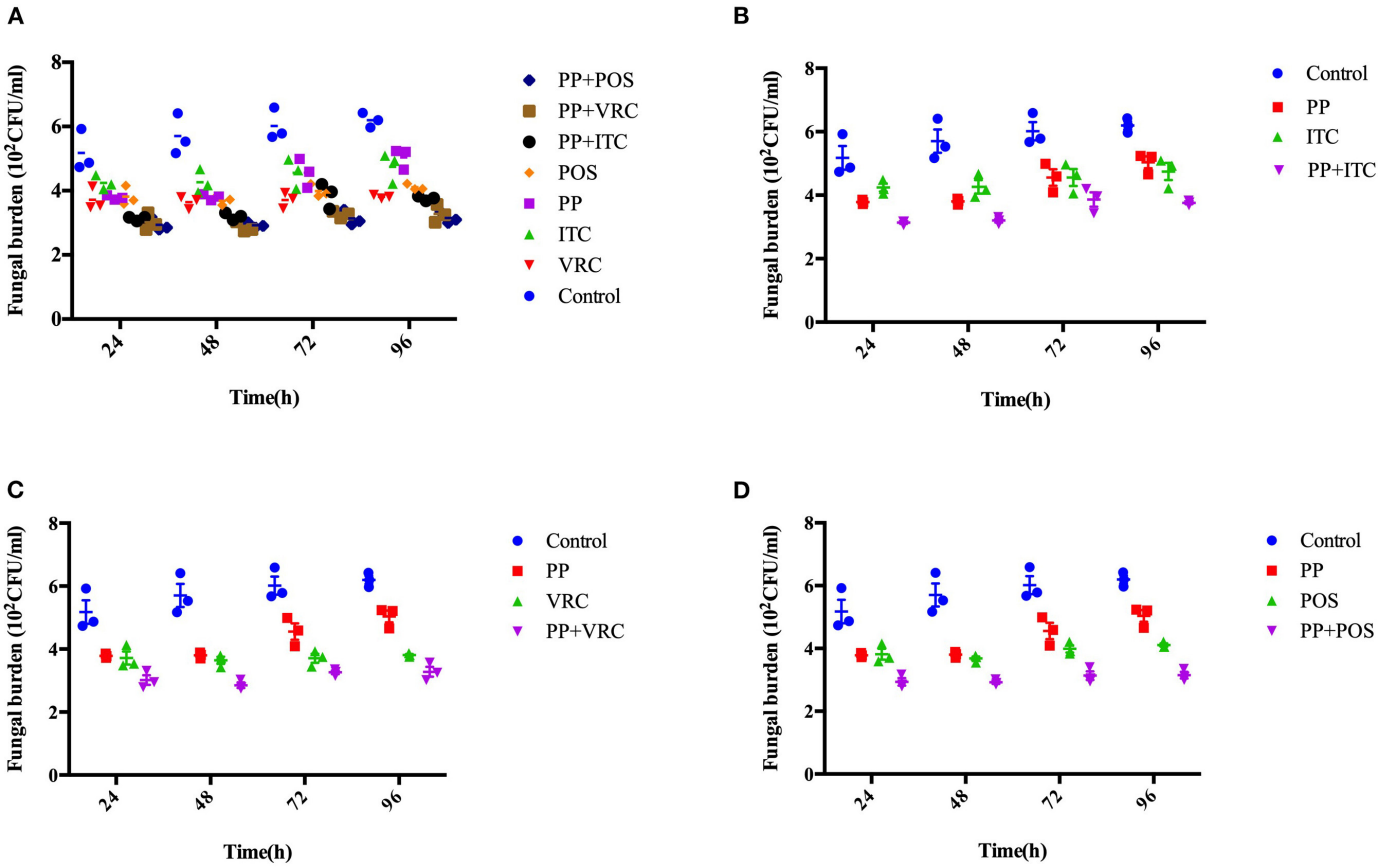
Fungal burden determination. Effect of drug combination on larval burdens of *E. dermatitidis*. All larvae were infected with 1 × 10^7^ cells/larva. **(A)** All groups. **(B)** Control group, PP group, ITC group, and PP with ITC group; **(C)** Control group, PP group, VRC group, and PP with VRC group; **(D)** Control group, PP group, POS group, and PP with POS group. The fungal burden of larva treated with PP-ITC and PP-VRC were significantly lower compared to ITC alone and VRC alone group (*P* < 0.01), respectively.

The fungal burden in larvae was determined by recovering fungal cells from the larvae infected with *E. dermatitidis*. The number of CFUs in larvae were increased over the time of infection (Figure 4). All treated groups exhibited significant lower CFUs compared to control group (*P* < 0.001). Infected larvae that were treated with azoles in combination with PP exhibited lower fungal burden than azole or PP alone group. Notably, the PP-ITC and PP-VRC combination significantly decreased CFU number compared to ITC and VRC alone group, respectively (*P* < 0.01).

### Histopathology Study

**Figure 5:**
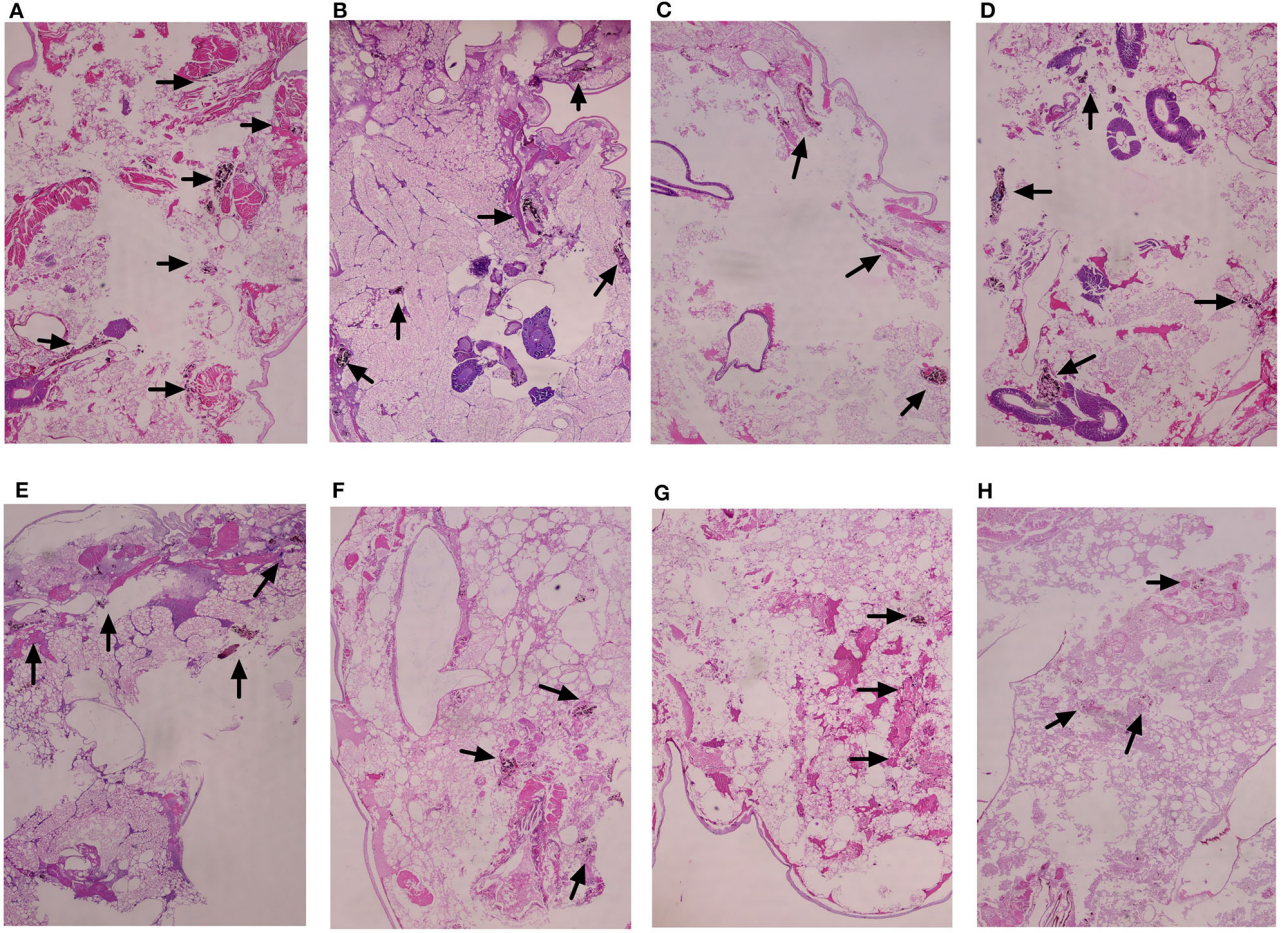
Histopathology study of infected *G. mellonella* treated with different drugs. **(A)** Untreated controls group; **(B)** PP treated group; **(C)** ITC treated group; **(D)** VRC treated group; **(E)** POS treated group; **(F)** PP with ITC treated group; **(G)** PP with VRC treated group; **(H)** PP with POS treated group. Treatment with PP or azoles alone **(B–E)** decreased yeast cells compared to control groups that received conidial suspension only **(A)**. In addition, the combination of PP and azoles decreased more formation of yeasts **(F–H)**. The group that received conidial suspension only **(A)** has higher levels of infection than any other group.

Histopathologic staining of larvae infected with *E. dermatitidis* and treated with different drugs was performed. The differences in shape and cytoplasmic staining were detected. *E. dermatitidis* yeast cell formed clusters within infected larvae. Treatment with PP or azoles alone (Figures 5B–E) decreased yeast cells compared to control groups that received conidial suspension only (Figure 5A). In addition, the combination of PP and azoles further decreased formation of yeasts (Figures 5F–H). The group that received conidial suspension only (Figure 5A) has higher levels of infection than any other group.

### Effect of PP Alone and Combined With Azoles on Apoptosis of *E. dermatitidis* Cells

**Figure 6:**
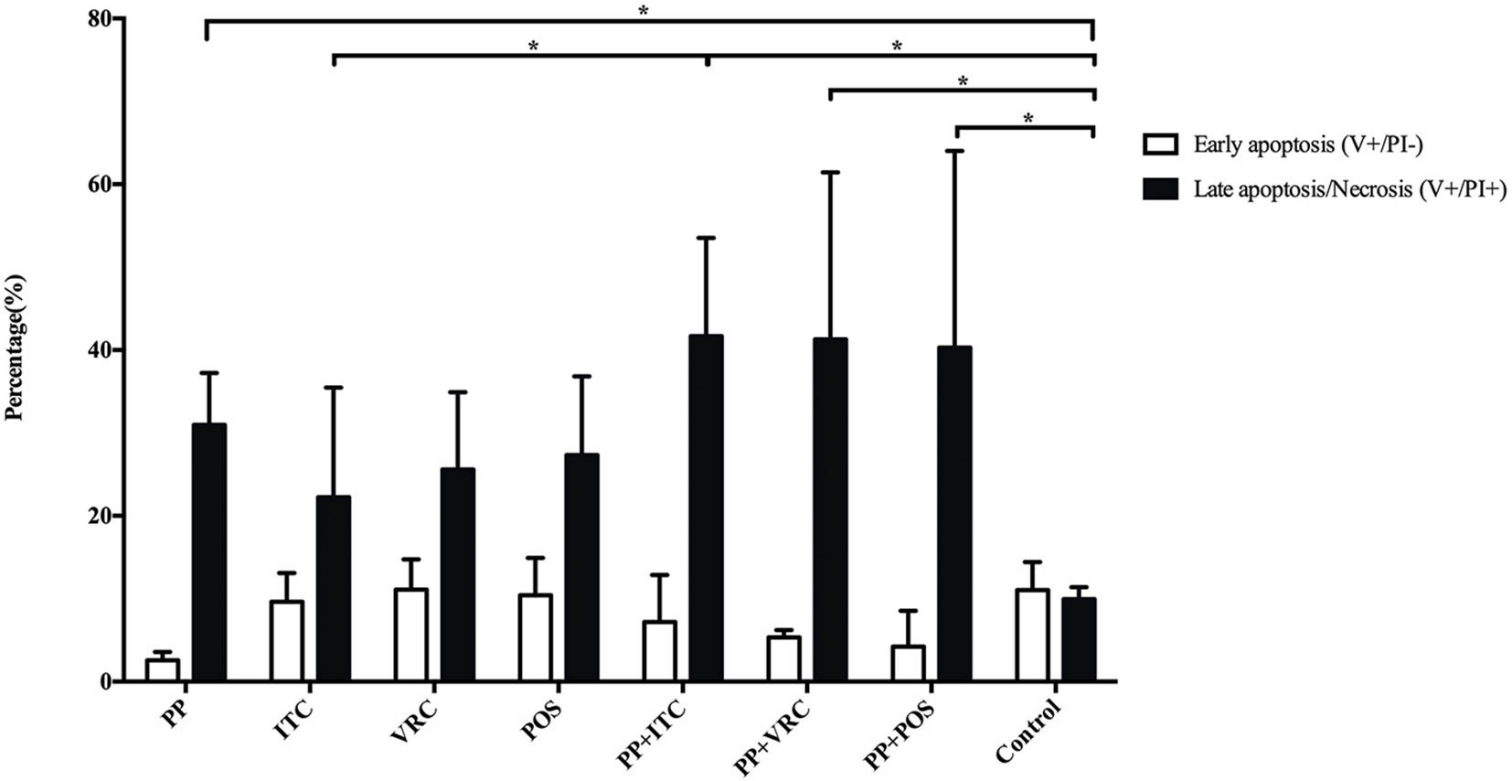
Apoptosis assay of *E. dermatitidis* cells. PP alone or combined with azoles significantly (*P* < 0.05) increased late apoptosis or necrosis of *E. dermatitidis* cells, compared to control group. The incidence of late apoptosis or necrosis of *E. dermatitidis* cells in the group treated with PP combined with ITC was significantly (*P* < 0.05) higher than the group treated with ITC alone. (*P < 0.05).

There is no significant difference in early apoptosis of *E. dermatitidis* cells among different intervention groups. However, PP alone or combined with azoles significantly (*P* < 0.05) increased late apoptosis or necrosis of *E. dermatitidis* cells, compared to control group (Figure 6). In addition, the incidence of late apoptosis or necrosis of *E. dermatitidis* cells in the group treated with PP combined with ITC was significantly (*P* < 0.05) higher than the group treated with ITC alone (Figure 6).

### Effect of PP Alone and Combined With Azoles on the Expression of *TOR* and *HSP90*

**Figure 7:**
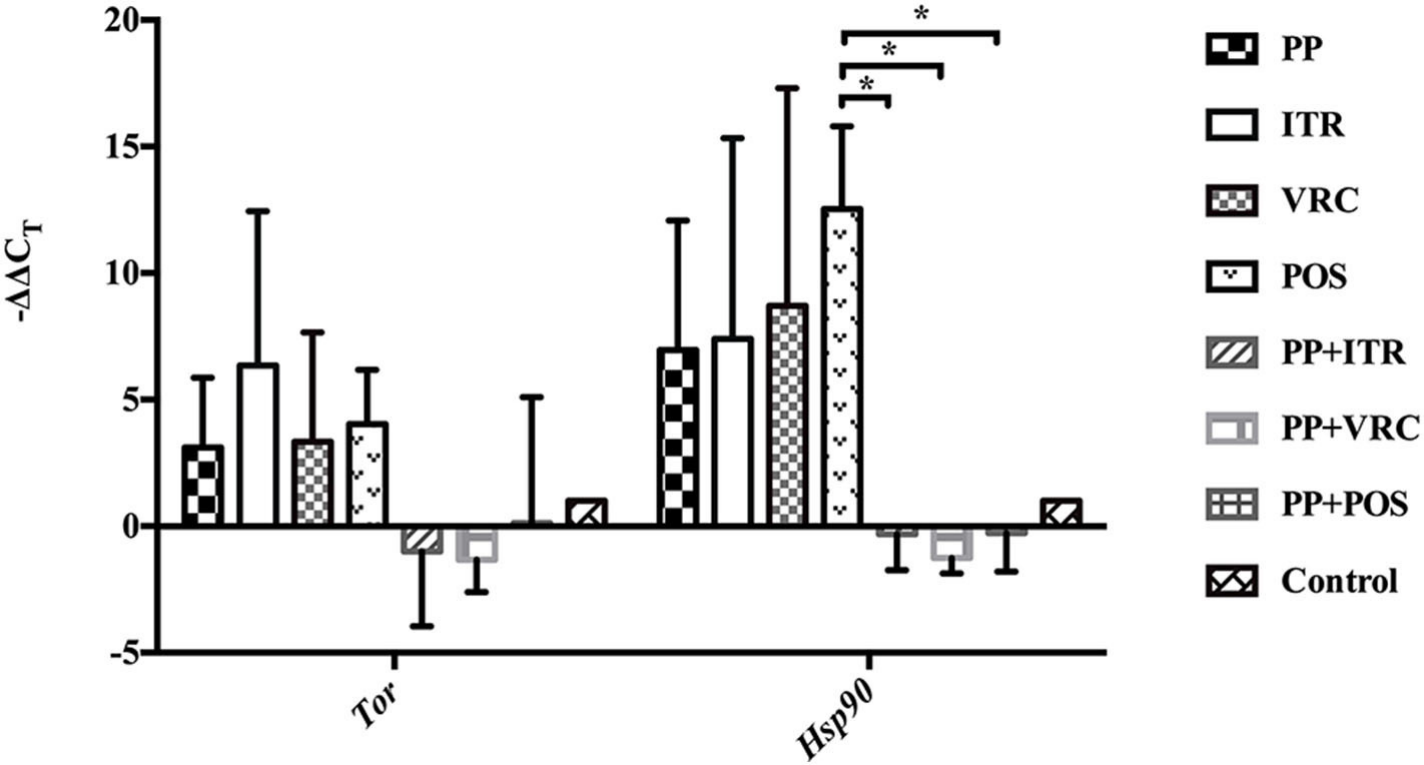
RT-qPCR assay of the expression of *TOR* and *HSP90*. PP or azole alone effectively induced the expression of both *TOR* and *HSP90*. On the contrary the *TOR* and *HSP90* expression was down-regulated when PP was combined with azoles, in comparison with PP alone or azole alone group. Notably, the mRNA level of both gene is effectively repressed in presence of PP and POS combination compared to POS group (*P < 0.05).

PP or azoles alone up-regulated the expression of both *TOR* and *HSP90* compared to control group. However, the combination of PP with azoles down-regulated the *TOR* and *HSP90* expression in comparison with PP alone or azole alone group (Figure 7). Notably, the combination of PP with POS group significantly decreased the expression level compared to POS alone group (*P* < 0.05).

### Effect of PP on the Efflux of Rhodamine 6G

**Figure 8:**
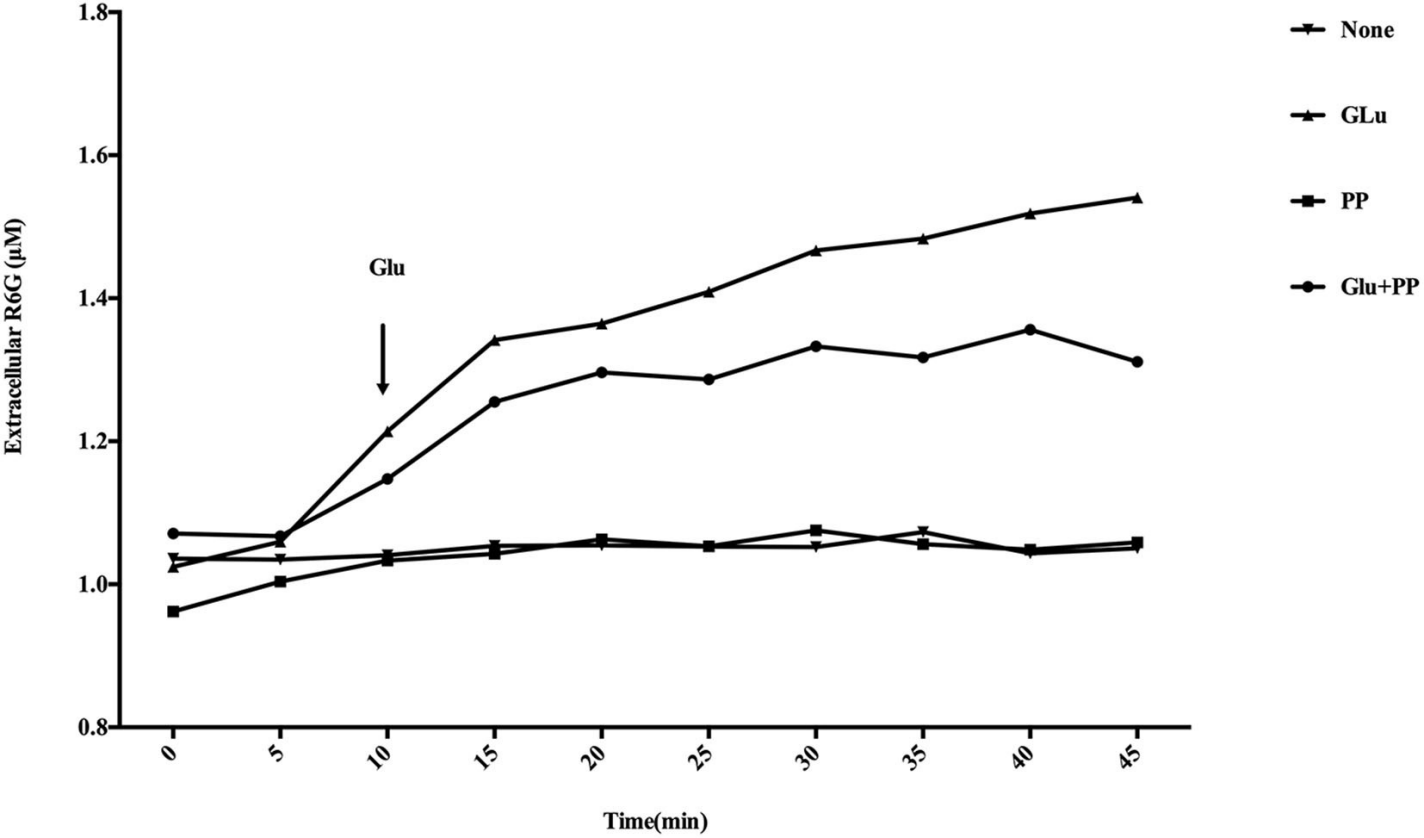
Effect of PP on R6G efflux in *E. dermatitidis*. Marked increase in measured extracellular R6G was observed when glucose was added to the conidial suspensions. There was a significant (*P* < 0.05) decrease in measured extracellular R6G concentrations following the addition of glucose and PP compared with conidial suspensions provided only glucose. No increase in measured extracellular R6G concentrations when PP was added to the conidial suspensions without glucose.

As shown in Figure 8, there was a marked increase in measured extracellular R6G concentrations when glucose was added to the conidial suspensions. However, no R6G efflux occurred when conidial suspensions were maintained for another 35 min in the absence of glucose, suggesting that the energy-dependent efflux pump activity is enhanced in the presence of glucose. There was a significant (*P* < 0.05) decrease in measured extracellular R6G concentrations following the addition of glucose and PP compared with conidial suspensions provided only glucose, suggesting PP has the ability to inhibit the efflux of R6G. There was no increase in measured extracellular R6G concentrations when PP was added to the conidial suspensions without glucose.

## Discussion

*E. dermatitidis* infections in immunosuppressive patients are most often in the form of phaeohyphomycosis (Matsumoto et al., 1993). There is no standard approach for the treatment of phaeohyphomycosis. Broad-spectrum azoles are currently the mainstay of therapy (Arcobello and Revankar, 2020). However, clinically phaeohyphomycosis often shows poor response to current antifungal modalities (Arcobello and Revankar, 2020). Multimodal therapy is often needed. With respect to antifungals, combination therapies with novel compounds and azoles, which has the potential to expand antimicrobial spectrum, improve treatment efficacy, and reduce side effects, could be an effective solution for recalcitrant fungal infections.

Previous study have demonstrated that PP potentiated the activity of fluconazole against fluconazole-resistant *C. albicans* (Chen et al., 2015). In our previous study, PP exerted modest antifungal activity alone (MIC 2 μg/ml) against *E. dermatitidis*. When PP was combined with POS, ITC, and VRC, synergism against 18 (100%), 16 (88.9%), and 9 (50%) strains of planktonic *E. dermatitidis* were observed, respectively (Gao et al., 2018). The effective working concentration ranges of PP in the combinations were as low as 0.125–0.5 μg/ml (0.1–0.4 μM) (Gao et al., 2018), demonstrating that the combination of PP and azoles could be a promising antifungal strategy.

In the present study, we first evaluated the *in vitro* effect of PP alone and combined with azoles against biofilms of *E. dermatitidis*. Synergism of PP with ITC, VRC, and POS were observed against 16 (88.9%), 9 (50%), and 13 (72.2%) strains of *E. dermatitidis* biofilms. The effect of PP alone on the growth of planktonic cells and biofilm formation of *E. dermatitidis* were further investigated. In accordance with *in vitro* susceptibilities (Gao et al., 2018), PP alone at concentration of 2 μg/ml resulted in significant restriction of growth of planktonic *E. dermatitidis*. Higher concentration of 4 μg/ml resulted in more significant inhibition of growth (Figure 1). Similarly, increasing concentrations of PP resulted in more reduction of biofilm formation (Figure 2). PP alone exhibited *in vitro* antifungal effect against both planktonic and biofilms of *E. dermatitidis*. Further, we observed the *in vivo* antifungal activity of PP alone and combined with azoles against *E. dermatitidis* infection using *G. mellonella* model, which showed similar immune response to mammals (Vilcinskas, 2011; Favre-Godal et al., 2014). PP alone exhibited comparable antifungal effect as azoles alone *in vivo*. The combination of PP and VRC or POS acted synergistically against *E. dermatitidis* infections. Fungal burden determination revealed that PP combined with ITC or POS significantly decreased CFU numbers in larvae compared to ITC or POS alone group, respectively. In addition, microscopic observation revealed fewer and smaller infected area on the histological tissue of *G. mellonella* in the combined group, confirming the correlation of the antifungal activity with the degree of tissue damage.

Furthermore, we investigated the possible antifungal mechanisms of PP alone and combined with azoles. Apoptosis has been implicated as a underlying mechanism of antifungal combination induced cell death in *Mucorales* (Shirazi and Kontoyiannis, 2013a,b). POS or ITC in combination with tacrolimus or mitochondrial respiratory pathways inhibition rendered *Mucorales* exquisitely sensitive to treatment with triazoles via apoptotic death (Shirazi and Kontoyiannis, 2013a,b). In the present study, apoptosis was measured by PI/Annexin V staining. Increasing early apoptosis were induced in azole alone groups, although without statistical significance. In contrary, PP alone result in slightly decreasing early apoptosis. Notably, PP alone and combined with azoles significantly (*P* < 0.05) increased late apoptosis or necrosis of *E. dermatitidis* cells, compared to control group. In addition, PP combined with ITC induced significantly more late apoptosis or necrosis of *E. dermatitidis* cells than ITC alone group. It's known that chemical/antifungal agents such as amphotericin B, acriflavin, and tunicamycin are able to cause both apoptosis and necrosis of fungal yeast cells (Eisenberg et al., 2010). Mitochondria plays a pivotal role during execution of apoptotic cell death, and also contributes to necrotic cell death in fungal cells (Eisenberg et al., 2010). Apoptosis is associated with mitochondrial impairment via caspase pathway, while serine/threonine kinases are implicated in necrotic cell death (Eisenberg et al., 2010). Previously, PP has been demonstrated to induce apoptosis of cancer cells via mitochondrial function impairment (Xiao et al., 2016; Momtazi-Borojeni et al., 2017). Therefore, we suspected that PP combined azoles might induced apoptotic or necrotic cell death of *E. dermatitidis* cells via mitochondrial respiration inhibition.

The TOR signaling pathway and calcineurin-Hsp90 signaling pathway are both essential and highly conserved among eukaryotes. Extensive investigations have implicated that the TOR signaling cascade is a central controller of cell growth in eukaryotes (Crespo and Hall, 2002). The TOR pathway regulates cellular responses to nutrients, including translation, transcription, proliferation, autophagy, morphogenesis, lipid homeostasis, ribosome biogenesis, and cellular aggregation in yeast cells, which have implicated important roles in virulence and pathogenicity (Crespo and Hall, 2002; Madeira et al., 2015). TOR kinases are the central elements of TOR signaling cascade. Calcineurin-Hsp90 pathway also governs multiple crucial process of fungal physiology, including virulence morphogenesis, and antifungal susceptibilities (Juvvadi and Steinbach, 2015). Hsp90, one of the most highly connected centers in cellular networks, interacts with an approximated 10% of the *S. cerevisiae* proteome (Zhao et al., 2005; McClellan et al., 2007). In pathogenic fungi, Hsp90 has been implicated to govern crucial stress responses and cell wall repair mechanisms (Juvvadi et al., 2014; Veri and Cowen, 2014; Lamoth et al., 2016). Hsp90 promoted resistance to azoles and echinocandins of pathogenic fungi via controlling the activity of its client proteins (Cowen and Lindquist, 2005; Cowen et al., 2009; Singh et al., 2009). Genetic compromise of Hsp90 reduces and abrogates the virulence of *C. albicans* and *A. fumigatus* in murine models, respectively, and potentiates the treatment effect of caspofungin and fluconazole in invasive candidiasis (Cowen et al., 2009; Singh et al., 2009; Lamoth et al., 2014). In addition, Hsp90 plays a key role in the dispersion of biofilm and antifungal resistance (Robbins et al., 2011). In the present study, the expression of *TOR* and *HSP90* were investigated. Intriguingly, the expression of both *TOR* and *HSP90* was effectively induced by PP or azole alone. This upregulated expression of *HSP90* was slightly higher than that of *TOR*. On the contrary, *TOR* and *HSP90* expression was down-regulated when PP was combined with azoles, in comparison with PP alone or azole alone group. Notably, the mRNA level of both gene is effectively repressed in presence of PP and POS combination compared to POS group (*P* < 0.05). These results revealed that the mechanism of synergism between PP and azoles maybe relative to the inhibition of stress response and growth control related signaling pathways.

In addition, to evaluate the ability of PP to mediate efflux reversal, we measured extracellular R6G concentrations in the absence or presence of PP using spectrophotometry analysis. The results revealed that PP has the ability to inhibit the efflux of R6G (*P* < 0.05), indicating that inhibition of drug efflux pumps might be an underlying mechanism of the antifungal activity of PP and the synergistic effect between azoles and PP.

Previous study showed that pyrvinium strongly suppressed the growth of the i(5L) strain (aneuploidy) of *C. albicans* (Chen et al., 2015). Aneuploidy, an abnormal chromosome number, arises frequently when fungal pathogens interact with host during infection (Todd et al., 2017). Aneuploidy of *C. albicans* has been demonstrated to be associated with azole resistance (Selmecki et al., 2006). However, whether ploidy change occurs in *E. dermatitidis* is still unknown. Further investigations are needed to elucidate the possible change of ploidy in *E. dermatitidis*, and the probable role of aneuploidy in the antifungal effect of PP against *E. dermatitidis*.

To our knowledge, this study provides a substantial investigation on the effect and underlying mechanism of PP alone and combined with azoles against *E. dermatitidis*. The *in vitro* antifungal effect of PP alone or combined with azoles against *E. dermatitidis* were confirmed by *G. mellonella* infection model. The underlying mechanisms could be explained by inducing apoptosis/necrosis, inhibition of drug efflux pumps, and signaling pathways associated with stress response and growth control. These results indicated that PP could be a favorable antifungal agent and a promising synergist with azoles against *E. dermatitidis*. However, PP is not completely soluble in aqueous solutions and there is no measurable absorption of pyrvinium from the gastrointestinal tract (Smith et al., 1976), which limits the possible application of pyrvinium in systemic infections.

Therefore, more in-depth investigation into the mechanism of antifungal properties of pyrvinium and the innovation of novel formulation of pyrvinium might help establishing novel antifungal strategies.

## Data Availability Statement

The raw data supporting the conclusions of this article will be made available by the authors, without undue reservation.

## Ethics Statement

Written informed consent was obtained from the individual(s) for the publication of any potentially identifiable images or data included in this article.

## Author Contributions

LG and YS conceived and designed the study. MY and LY performed all the experiments. LG and YS analyzed the data and wrote the manuscript. JY and TZ provided general guidance and revised the manuscript. All authors contributed to the article and approved the submitted version.

## Conflict of Interest

The authors declare that the research was conducted in the absence of any commercial or financial relationships that could be construed as a potential conflict of interest.
